# Clinical efficacy of xenon versus propofol: A systematic review and meta-analysis

**DOI:** 10.1097/MD.0000000000010758

**Published:** 2018-05-18

**Authors:** Yimeng Xia, Hongwei Fang, Jindong Xu, Chenfei Jia, Guorong Tao, Buwei Yu

**Affiliations:** a Department of Anaesthesiology, Ruijin Hospital, Shanghai Jiaotong University School of Medicine; b Department of Anaesthesiology and Intensive Care Unit, Dongfang Hospital, Tongji University School of Medicine, Shanghai; c Department of Anaesthesiology, Guangdong Cardiovascular Institute & Guangdong General; Hospital, Guangdong Academy of Medical Sciences, Guangdong Province, People's Republic of China.

**Keywords:** general anesthetics, meta-analysis, propofol, randomized controlled trials, xenon

## Abstract

**Background:**

Interest in the anesthetic use of xenon, a noble gas, has waxed and waned for decades, and the clinical effects of xenon are still debated. We performed a meta-analysis to compare the clinical efficacy of xenon with that of propofol.

**Methods:**

Electronic searches were performed through December 2017 using various databases, including PubMed, Embase, and the Cochrane Library. We identified thirteen trials that included a total of 817 patients.

**Results:**

Patients treated with xenon had a lower bispectral index (BIS) (weighted mean difference (WMD): −6.26, 95% confidence interval (CI): −11.33 to −1.18, *P = *.02), a higher mean arterial blood pressure (MAP) (WMD: 7.00, 95% CI: 2.32–11.68, *P = *.003) and a lower heart rate (HR) (WMD: −9.45, 95% CI: −12.28 to −6.63, *P* < 0.00001) than propofol-treated patients. However, there were no significant differences between the 2 treatment groups in the effects of nondepolarizing muscular relaxants, the duration spent in the postanesthesia care unit (PACU) (WMD: −0.94, 95% CI: −8.79–6.91, *P = *.81), or the incidence of perioperative complications [assessed using the outcomes of postoperative nausea and vomiting (PONV) (relative risk (RR): 2.01, 95% CI: 0.79–5.11, *P = *.14), hypotension (RR: 0.62, 95% CI: 0.27 to 1.40, *P = *.25), hypertension (RR: 1.27, 95% CI: 0.73–2.21, *P = *.39) and bradycardia (RR: 1.00, 95% CI: 0.36–2.74, *P* = 1.00)].

**Conclusion:**

In this meta-analysis of randomized controlled trials, we found that xenon treatment resulted in a higher MAP, a lower HR, and a smaller BIS index than treatment with propofol.

## 1 Introduction

Xenon, which was first used as a general anesthetic in 1951,^[1]^ is an alternative to currently used anesthetics. While xenon has a high cost, it also has many advantages over other anesthetics, such as low blood-gas and brain-blood coefficients, rapid induction and recovery, almost no respiratory, hepatic or renal toxicity, stable hemodynamics, and effective neuroprotective and environmentally friendly properties.^[2]^

During the past decade, a number of randomized controlled trials (RCTs) have been published that have compared the clinical efficacies of xenon and other volatile or intravenous anesthetics.^[3–20]^Although one meta-analysis^[2]^ has summarized these individual studies, it contained some specific errors and failed to include important clinical data related to propofol.

Propofol, one of the most widely used intravenous anesthetics, has a fast induction and recovery, is associated with short stays in postanesthesia care units (PACUs), and has few effects on patient movement.^[21]^ When used inappropriately, propofol can cause hypotension, bradycardia, injection pain, and respiratory depression.^[21,22]^ Clinically, the bispectral index (BIS) is a valuable method for monitoring the anesthetic effect of propofol.^[23]^

By carefully analysing the available data, we performed a meta-analysis of published RCTs to compare the clinical efficacies of xenon and propofol.

## 2 Methods

### 2.1 Search strategy

The present study was performed by searching the PubMed, Embase, and Cochrane Library databases to retrieve relevant studies that were published through December 2017 and described clinical comparisons between xenon and propofol. All analyses were based on previous published studies, thus no ethical approval and patient consent are required. The search was restricted to articles published in the English language. The initial search process involved the terms (“Xenon”) and (“propofol” or “ICI 35868,” or “2,6-diisopropylphenol”) and (“anesthesi∗” or “anaesthesi∗”). Moreover, we excluded 2 retracted articles.^[24,25]^ The authors were not contacted for any additional information, and our search results did not include any unpublished studies.

### 2.2 Study selection

**Figure 1:**
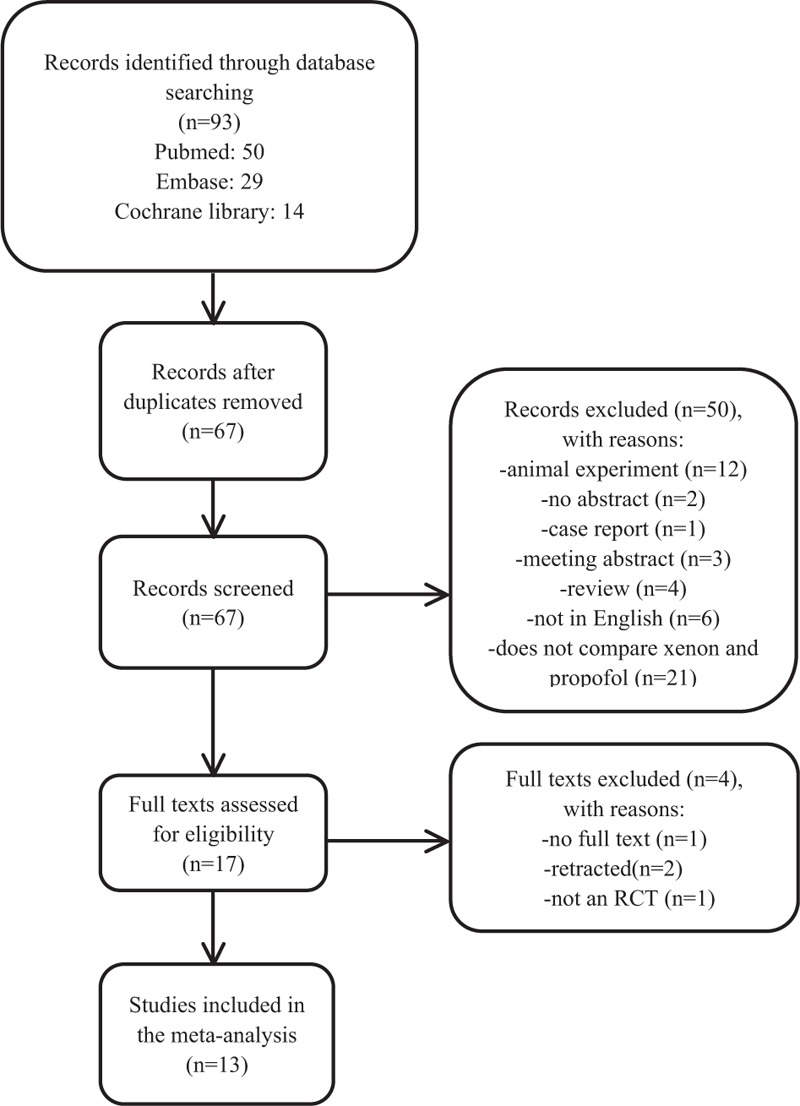
The process used to perform the literature search.

The first step was to screen potential abstracts and titles. Full-text reviews were performed in the second round. We defined the trials as eligible if they conformed to the following inclusion criteria: comparisons between xenon and propofol; RCTs; the outcomes of interest were time in the PACU, the influence of xenon on nondepolarizing muscular relaxants, BIS index, hemodynamic effects, and side effects, such as hypotension, bradycardia, hypertension and postoperative nausea and vomiting (PONV). For more details, see Figure 1.

### 2.3 Data collection and risk of bias

YX and HF independently performed the electronic search and data extraction. Arguments were settled by a third investigator (CJ). The data were extracted according to the following standard form: last name of the first author, publication year, number of patients, the dosage and time of the anesthetics, and the type of surgery.

With the help of the Cochrane collaboration's tools, we established a table to determine “risk of bias” of the selected trials according to the following 6 parameters: adequate sequence generation, allocation concealment, blinding of participants and personnel, blinding of outcome assessments, incomplete outcome data, and selective outcome reporting. We labeled each parameter as “low”, “high,” or “unclear” to clarify the risk of bias.

### 2.4 Statistical analysis

We used Review Manager 5.3 software (Cochrane Collaboration, Copenhagen) to perform all statistical analyses. Dichotomous outcomes are presented as relative risks (RRs), and continuous data are shown as the weighted mean difference (WMD). Both include 95% confidence intervals (CIs). If significant heterogeneity was detected, the pooled estimates were calculated using a random-effects model. Otherwise, a fixed effects model was used, and *z* tests were used to assess the effects. Continuous results are shown as the mean ± standard deviation (SD), and the chi-square test and *I*^2^ statistic were used to test for heterogeneity among the trials. *P* values < .05 were regarded as statistically significant.

## 3 Results

Figure 1 shows an outline of the literature search and selection process. After duplicates were deleted, 67 studies were identified. After the article titles and abstracts were examined, 17 studies were included. Moreover, 2 retracted studies,^[24,25]^ one study for which the full text was lost^[14]^ and one study that was not an RCT,^[26]^ were excluded. Finally, 13 studies that included a total of 817 patients were selected for the analysis.^[3–10,13,15–17,20]^ The evaluated trials included reports that were published through December 2017. The baseline characteristics of the pooled studies are summarized in Table 1, which includes patient age and the American Society of Anesthesiologists (ASA) status, which anesthetic drugs were administered and what type of surgery was used, and the results that are relevant to the analysis. Table 2 includes the risk assessment.

**Table 1:**
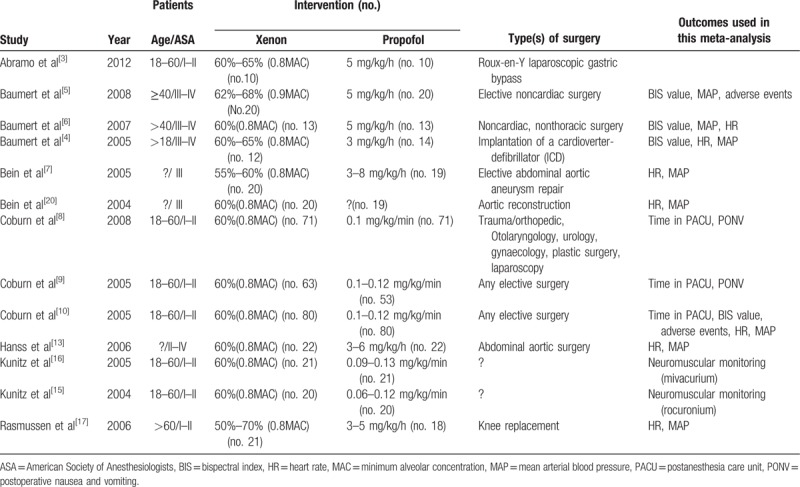
Basic characteristics of included studies.

**Table 2:**
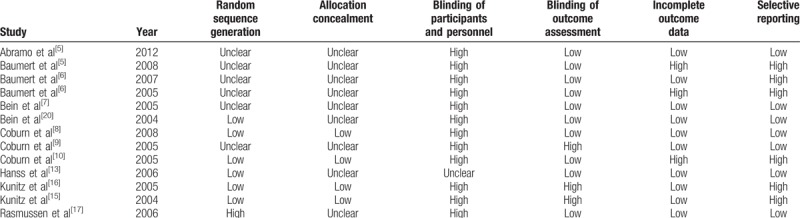
Risk of bias in included studies.

### 3.1 Primary outcome

#### 3.1.1 BIS values

**Figure 2:**
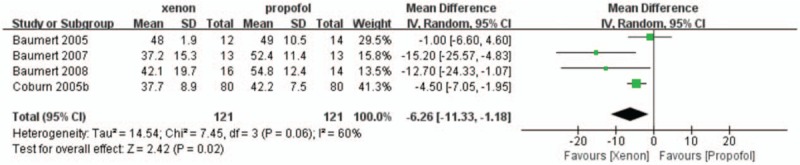
The BIS index in the xenon group versus that in the propofol group. IV = inverse variance, random = random effect, 95% CI = 95% confidence interval.

BIS values were assessed in 4 studies.^[4–6,10]^ All studies showed that there was a lower index in the xenon group than in the propofol group. The pooled mean difference between the xenon and propofol groups was calculated (WMD: −6.26, 95% CI: −11.33 to −1.18, *P = *.02; Fig. 2) and was significantly different between the 2 groups.

#### 3.1.2 Influence on nondepolarizing muscular relaxants

Two studies assessed how nondepolarizing muscle relaxation was affected by xenon.^[15,16]^ Both studies included the time of onset, duration (T_25_), clinical recovery (T_25–0.8_), and recovery index (T_25–75_). No significant differences were observed between the xenon group and the propofol group (Table 3).

**Table 3:**
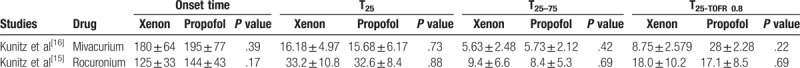
Influence of xenon on nondepolarizing neuromuscular relaxants.

#### 3.1.3 PACU length

**Figure 3:**
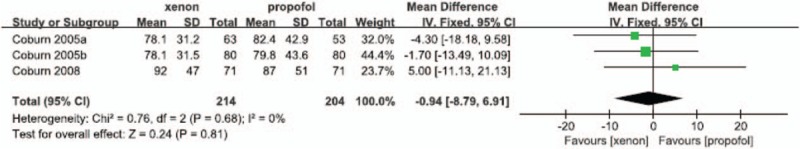
PACU stay length in the xenon group versus that in the propofol groups. IV = inverse variance, fixed = fixed effect, 95% CI = 95% confidence interval.

The data are expressed as the mean (±SD) duration time, and the length of stay in the PACU after xenon treatment was evaluated in 3 trials.^[8–10]^ No significant difference was found between the xenon and propofol groups (WMD: −0.94, 95% CI: −8.79 to 6.91, *P = *.81; Fig. 3).

### 3.2 Secondary outcomes

#### 3.2.1 Perioperative complications

**Figure 4:**
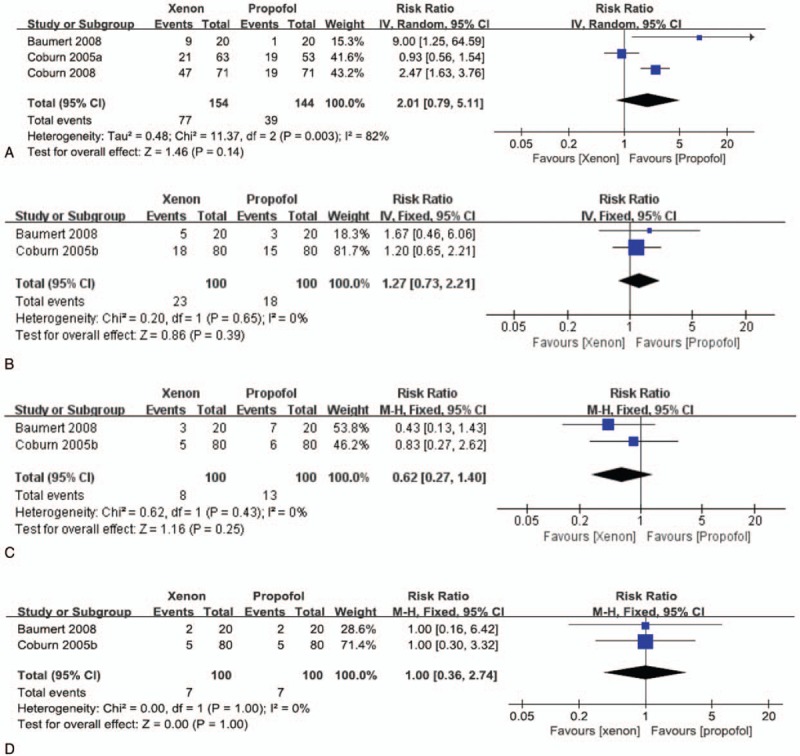
The incidence of perioperative complications in the xenon group versus that in the propofol group: (A) postoperative nausea and vomiting (PONV), (B) hypertension, (C) hypotension and (D) bradycardia. PONV = postoperative nausea and vomiting.

We analysed three studies^[5,8,9]^ that compared PONV and 2 studies^[5,10]^ that investigated hypotension, hypertension, and bradycardia between xenon-and propofol-treated patients. There was no significant difference in the incidence of PONV (RR: 2.01, 95% CI: 0.79–5.11, *P = *.14; Fig. 4A), hypertension (RR: 1.27, 95% CI: 0.73–2.21, *P = *.39; Fig. 4B), hypotension (RR: 0.62, 95% CI: 0.27–1.40, *P = *.25; Fig. 4C), or bradycardia (RR: 1.00, 95% CI: 0.36–2.74, *P* = 1.00; Fig. 4D) between the groups.

#### 3.2.2 Hemodynamic changes

**Figure 5:**
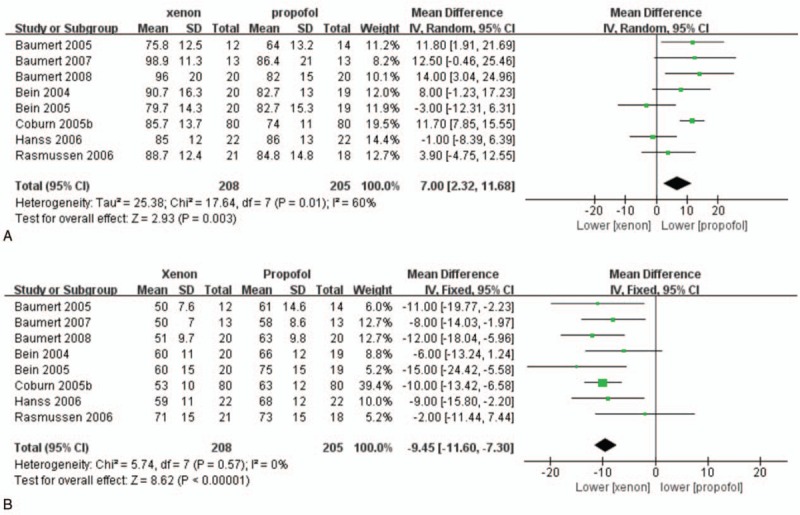
Perioperative hemodynamics in the xenon group versus those in the propofol groups: (A) MAP and (B) HR. HR = heart rate, MAP = mean arterial blood pressure.

Eight papers that included a total of 413 patients reported values for mean arterial blood pressure (MAP) and heart rate (HR).^[4–7,10,13,17,20]^ Higher MAP (WMD:7.00, 95% CI: 2.32–11.68, *P = *.003; Fig. 5A) and lower HR (WMD: −9.45, 95% CI: −12.28 to −6.63, *P* < .00001; Fig. 5B) values were observed in the xenon group than in the propofol group.

## 4 Discussion

The present meta-analysis included 13 studies. Our objective was to compare the BIS index, the effects on nondepolarizing muscular relaxants, the length of stay in the PACU, hemodynamic changes and perioperative complications between patients who were administered xenon versus propofol as a general anesthetic.

Our analysis revealed that patients who were administered xenon had a higher MAP, lower HR, and lower BIS index than patients administered propofol. However, there was no difference between the 2 treatment groups in the effects of the treatments on nondepolarizing muscular relaxants, the length of stay in the PACU or perioperative complications.

Similar to a previous meta-analysis,^[2]^ we compared hemodynamic changes, perioperative complications and PACU stay length between the xenon and propofol groups. Although some data corrections were made, such as the correction that 20 and not 13 patients were in each group that was used to compare the incidences of PONV,^[2,8]^ we reached similar conclusions. Compared to a previous analysis,^[2]^ we added one more study^[15]^ and detected additional vital data, including BIS values and the influence of the anesthetics on the activity of neuromuscular blockers.

When applied as an anesthetic, xenon is thought to act by antagonizing glutamatergic neurotransmission at *N*-methyl-D-aspartate (NMDA) receptors.^[27]^ Electroencephalogram (EEG)-based indices such as BIS are now commonly used to determine the state of hypnosis during general anesthesia, and this practice allows the anaesthesiologist to decrease both the consumption of anesthetics and the incidence of patient awareness.^[28]^ However, since ketamine (another NMDA-receptor antagonist) has been shown to increase BIS values, it seems paradoxical that the anesthesia level is deepened by additional anesthetic agents.^[29–31]^ Whether BIS levels are always a good indicator for anesthetics acting via NMDA receptors remains uncertain. We therefore evaluated the performance of anesthesia depth monitors by comparing BIS values between the 2 groups across 4 clinical trials.^[4–6,10]^ Our results showed that the BIS value was significantly lower in the xenon group than in the propofol group (propofol acts by potentiating γ-aminobutyric acid A [GABAA] receptor activity).^[32]^ A possible explanation for this result may be that different mechanisms of anesthetic action are used to produce unconsciousness. Additionally, the lower BIS values that are observed after xenon treatment than after propofol treatment are due to data averaging and technical delay behind the true EEG processes owing to the rapid emergence from xenon anesthesia. Moreover, combined treatment with a GABAergic drug like propofol or sevoflurane may change the EEG pattern and interfere with NMDA antagonist anesthetics to produce inaccurate BIS values as with ketamine. These results suggest that monitoring the BIS index may not be suitable when assessing the depth of xenon-induced anesthesia, although it may be adequate for assessing the effect of propofol.

Xenon allows patients to rapidly emerge and recover from anesthesia because of its extremely low blood-gas solubility.^[15,33]^ Two included RCTs showed that xenon did not affect nondepolarizing muscle relaxation. Moreover, we did not observe any differences in the length of stay in the PACU, which is another recovery index. These results support the claim that xenon is clinically safe and has good efficacy.

We acknowledge that there are limitations to the present meta-analysis. First, only articles that were published in English were retrieved, and the data for most of the comparisons examined in this study were obtained from 4 or fewer studies. Thus, our conclusions may be based on relatively small numbers of patients. Second, there was heterogeneity in some study characteristics, including the types of surgery, patient populations, and perioperative opioid consumption. Finally, the influence of publication bias should be recognized.

In conclusion, xenon has been demonstrated to have good clinical efficacy and safety with regard to recovery time, influence on neuromuscular blockers, and postoperative complications, and it may therefore be a good alternative to general anesthetics. In addition, clinicians must take the higher MAP, lower HR, and lower BIS values associated with xenon into consideration when using this drug instead of propofol as an anesthetic.

## Author contributions

**Conceptualization:** Guorong Tao.

**Data curation:** Yimeng Xia, Hongwei Fang, Chenfei Jia.

**Formal analysis:** Yimeng Xia, Guorong Tao.

**Methodology:** Chenfei Jia.

**Software:** Jindong Xu.

**Writing – original draft:** Yimeng Xia, Hongwei Fang, Jindong Xu.

**Writing – review & editing:** Yimeng Xia, Buwei Yu.

## References

[1] Cullen SC Gross EG The anesthetic properties of xenon in animals and human beings, with additional observations on krypton. Science 1951;113:580–2. 14834873 10.1126/science.113.2942.580

[2] Law LS LoE A Gan TJ Xenon anesthesia: a systematic review and meta-analysis of randomized controlled trials. Anesth Analg 2016;122:678–97. 26273750 10.1213/ANE.0000000000000914

[3] Abramo A Di Salvo C Baldi G Xenon anesthesia reduces TNFα and IL10 in bariatric patients. Obes Surg 2012;22:208–12. 21559793 10.1007/s11695-011-0433-y

[4] Baumert JH Falter F Eletr D Xenon anaesthesia may preserve cardiovascular function in patients with heart failure. Acta Anaesthesiol Scand 2005;49:743–9. 15954952 10.1111/j.1399-6576.2005.00662.x

[5] Baumert JH Hein M Hecker KE Xenon or propofol anaesthesia for patients at cardiovascular risk in non-cardiac surgery. Br J Anaesth 2008;100:605–11. 18344556 10.1093/bja/aen050

[6] Baumert JH Hein M Hecker KE Autonomic cardiac control with xenon anaesthesia in patients at cardiovascular risk. Br J Anaesth 2007;98:722–7. 17468494 10.1093/bja/aem083

[7] Bein B Turowski P Renner J Comparison of xenon-based anaesthesia compared with total intravenous anaesthesia in high risk surgical patients. Anaesthesia 2005;60:960–7. 16179039 10.1111/j.1365-2044.2005.04326.x

[8] Coburn M Kunitz O Apfel CC Incidence of postoperative nausea and emetic episodes after xenon anaesthesia compared with propofol-based anaesthesia. Br J Anaesth 2008;100:787–91. 18397921 10.1093/bja/aen077

[9] Coburn M Kunitz O Baumert JH Patients’ self-evaluation after 4-12 weeks following xenon or propofol anaesthesia: a comparison. Eur J Anaesthesiol 2005;22:870–4. 16225724 10.1017/S026502150500147X

[10] Coburn M Kunitz O Baumert JH Randomized controlled trial of the haemodynamic and recovery effects of xenon or propofol anaesthesia. Br J Anaesth 2005;94:198–202. 15531620 10.1093/bja/aei023

[11] Fahlenkamp AV Coburn M Rossaint R Comparison of the effects of xenon and sevoflurane anaesthesia on leucocyte function in surgical patients: a randomized trial. Br J Anaesth 2014;112:272–80. 24131665 10.1093/bja/aet330

[12] Fahlenkamp AV Krebber F Rex S Bispectral index monitoring during balanced xenon or sevoflurane anaesthesia in elderly patients. Eur J Anaesthesiol 2010;27:906–11. 20671557 10.1097/EJA.0b013e32833d1289

[13] Hanss R Bein B Turowski P The influence of xenon on regulation of the autonomic nervous system in patients at high risk of perioperative cardiac complications. Br J Anaesth 2006;96:427–36. 16500952 10.1093/bja/ael028

[14] Johnson BW Sleigh JW Kirk IJ High-density EEG mapping during general anaesthesia with xenon and propofol: a pilot study. Anaesth Intensive Care 2003;31:155–63. 12712778 10.1177/0310057X0303100203

[15] Kunitz O Baumert JH Hecker K Xenon does not prolong neuromuscular block of rocuronium. AnesthAnalg 2004;99:1398–401. 10.1213/01.ANE.0000132970.64413.E7 15502037

[16] Kunitz O Baumert JH Hecker K Xenon does not modify mivacurium induced neuromuscular block. Can J Anaesth 2005;52:940–3. 16251559 10.1007/BF03022055

[17] Rasmussen LS Schmehl W Jakobsson J Comparison of xenon with propofol for supplementary general anaesthesia for knee replacement: a randomized study. Br J Anaesth 2006;97:154–9. 16782975 10.1093/bja/ael141

[18] Stoppe C Fahlenkamp AV Rex S Feasibility and safety of xenon compared with sevoflurane anaesthesia in coronary surgical patients: a randomized controlled pilot study. Br J Anaesth 2013;111:406–16. 23578862 10.1093/bja/aet072

[19] Bronco A Ingelmo PM Aprigliano M Xenon anaesthesia produces better early postoperative cognitive recovery than sevoflurane anaesthesia. Eur J Anaesthesiol 2010;27:912–6. 20523212 10.1097/EJA.0b013e32833b652d

[20] Bein B Hanne P Hanss R Effect of xenon anaesthesia on accuracy of cardiac output measurement using partial CO2 rebreathing. Anaesthesia 2004;59:1104–10. 15479320 10.1111/j.1365-2044.2004.03897.x

[21] Fang H Yang L Wang X Clinical efficacy of dexmedetomidine versus propofol in children undergoing magnetic resonance imaging: a meta-analysis. Int J Clin Exp Med 2015;8:11881–9. 26550100 PMC4612785

[22] Sebel PS Lowdon JD Propofol: a new intravenous anesthetic. Anesthesiology 1989;71:260–77. 2667401

[23] Glass PS Bloom M Kearse L Bispectral analysis measures sedation and memory effects of propofol, midazolam, isoflurane, and alfentanil in healthy volunteers. Anesthesiology 1997;86:836–47. 9105228 10.1097/00000542-199704000-00014

[24] Höcker J Raitschew B Meybohm P Differences between bispectral index and spectral entropy during xenon anaesthesia: a comparison with propofol anaesthesia. Anaesthesia 2010;65:595–600. 20412149 10.1111/j.1365-2044.2010.06344.x

[25] Höcker J Stapelfeldt C Leiendecker J Postoperative neurocognitive dysfunction in elderly patients after xenon versus propofolanesthesia for major noncardiac surgery: a double-blinded randomized controlled pilot study. Anesthesiology 2009;110:1068–76. 19352169 10.1097/ALN.0b013e31819dad92

[26] Neukirchen M Schaefer MS Kern C Xenon does not increase heart rate-corrected cardiac QT interval in volunteers and in patients free of cardiovascular disease. Anesthesiology 2015;123:542–7. 26164300 10.1097/ALN.0000000000000764

[27] Maze M Preclinical neuroprotective actions of xenon and possible implications for human therapeutics: a narrative review. Can J Anaesth 2016;63:212–26. 26507536 10.1007/s12630-015-0507-8

[28] Laitio RM Kaskinoro K Särkelä MO Bispectral index, entropy, and quantitative electroencephalogram during single-agent xenon anesthesia. Anesthesiology 2008;108:63–70. 18156883 10.1097/01.anes.0000296106.52472.a6

[29] Hans P Dewandre PY Brichant JF Comparative effects of ketamine on bispectral Index and spectral entropy of the electroencephalogram under sevoflurane anaesthesia. Br J Anaesth 2005;94:336–40. 15591328 10.1093/bja/aei047

[30] Park KS Hur EJ Han KW Bispectral index does not correlate with observer assessment of alertness and sedation scores during 0.5% bupivacaine epidural anesthesia with nitrous oxide sedation. Anesth Analg 2006;103:385–9. 16861421 10.1213/01.ane.0000226090.13170.25

[31] Maksimow A Särkelä M Långsjö JW Increase in high frequency EEG activity explains the poor performance of EEG spectral entropy monitor during S-ketamine anesthesia. Clin Neurophysiol 2006;117:1660–8. 16807101 10.1016/j.clinph.2006.05.011

[32] Krasowski MD Jenkins A Flood P General anesthetic potencies of a series of propofol analogs correlate with potency for potentiation of gamma-aminobutyric acid (GABA) current at the GABA(A) receptor but not with lipid solubility. J Pharmacol Exp Ther 2001;297:338–51. 11259561

[33] Goto T Suwa K Uezono S The blood-gas partition coefficient of xenon may be lower than generally accepted. Br J Anaesth 1998;80:255–6. 9602599 10.1093/bja/80.2.255

